# The relationship between patellar lateralization diagnostic imaging markers and non-contact internal knee derangements

**DOI:** 10.1186/s13018-020-01661-2

**Published:** 2020-04-25

**Authors:** Kyle S. Stumetz, M. D. Gothard, Ronald F. Walser, Alan G. Greenwald, Wade W. Justice

**Affiliations:** 1 Yakima, USA; 2 East Canton, USA

**Keywords:** TT-TG, PT-PCL, Patellar instability, Patellar lateralization, Meniscus tears, Cruciate tears

## Abstract

**Background:**

To investigate differences in the tibial tubercle-trochlear groove (TT-TG) and patellar tendon-posterior cruciate ligament (PT-PCL) distances in symptomatic patients with non-contact internal knee derangements (IKD) and symptomatic patients with internally intact knees (control).

**Methods:**

A retrospective review of MRI studies was completed by comparing 78 patients with meniscal and ligamentous derangements of the knee to 63 internally intact knees (age range, 13 to 50 years). MRI findings were reviewed independently by two board-certified radiologists to assess for agreement. TT-TG and PT-PCL distances were measured on proton density-weighted axial images by two independent observers blinded to the MRI and arthroscopic findings. Independent *t* tests were used to determine differences in TT-TG distance between the internal derangement and control groups. Chi-square tests were used to compare categorical variables for distributional equality between study groups.

**Results:**

The mean TT-TG distance averaged across the two raters in the IKD group was 11.5 mm (95% confidence interval [CI], 10.6–12.4), compared to 8.3 mm (95% CI, 7.6–9.0) in the control group (*p* < 0.001). The mean PT-PCL distance similarly averaged across both raters was 20.6 mm (95% CI, 19.7–21.5) for the IKD group compared to 18.2 mm (95% CI, 17.2–19.2) for the control group (*p* < 0.001). Among the IKD group, there were 51 meniscal tears, 12 cruciate ligament tears, and 15 tears with a combination of meniscal and cruciate findings. IKD was significantly correlated with greater TT-TG distance (*p* < 0.001) and greater PT-PCL distance (*p* < 0.003) when compared with control.

**Conclusions:**

Increased TT-TG distances and PT-PCL distances are associated with both cartilaginous and ligamentous internal knee injuries in the present study, with TT-TG distances greater than the 12 mm representing a new threshold for concern.

## Background

The tibial tubercle-trochlear groove distance (TT-TG) and patellar tendon-posterior cruciate ligament (PT-PCL) distances are important measurements in the assessment of patellofemoral disorders [1–4]. In cases of patellar instability and recurrent dislocations, they have been used to assess the degree of patellar lateralization as well as to aid in establishing the most appropriate surgical approach for a given patient [5, 6]. Originally described on plain film radiographs and later adapted to CT and MRI, the TT-TG distance has been described as a radiographic analog to the quadriceps angle (Q angle) [7–9]. It has become an increasingly useful measurement given its excellent interrater reliability and ease of use. Additionally, a less commonly used measurement, but valued based upon its consistency regardless of knee position, the PT-PCL has been utilized to assess for lateralization of the patella and is similarly helpful in work-up of patients with patella-femoral dislocation [4].

In the context of a patellar dislocation, TT-TG values greater than 15 to 20 mm are widely considered to be abnormal [9–11], yet there is a lack of consensus in the literature regarding which TT-TG values fall within the normal range. The normal range has previously been reported to be as low as 8.2 ± 3.7 mm [12] and as high as 13.6 ± 8.8 mm [13] with many studies falling somewhere in the middle [2, 12–16]. Additionally, the aforementioned normal studies have been complicated by inconsistent inclusion and exclusion criteria. Given the primary focus on patellar dislocation in previous studies, the control groups have commonly included a variety of other internal knee derangements as long as the clinical history of patellar instability is absent. Cruciate ligament tears, as well as meniscal tears, are often included with internally intact knees to comprise the control TT-TG group [16–19]. Building on the findings of Saper et al. which raised the possibility of an association between internal derangement (other than a patella-femoral dislocation) and increased lateralization of the patella, this practice is being brought into question by the present study [16].

Thus, the goal of the present study was threefold as follows: to further define the normal TT-TG range in anatomically intact knees, to provide a more comprehensive stratification of the TT-TG measurement in the context of internal knee derangements, and to test the validity of these findings by measuring the PT-PCL distance given its consistency irrespective of knee positioning [17, 18]. Cruciate ligament tears as well as meniscal tears were the primary internal derangements accounted for.

## Methods

### Study population

Institutional review board approval and waiver of informed consent were obtained for this retrospective case-control study prior to the start of the study. Investigators identified 280 consecutive knee MRI examinations from subjects referred to the medical center imaging department for non-traumatic symptomatic knee pain, between January 1, 2017, and July 15, 2018***.*** The age range of 13–50 years and non-contact mechanism of injury were both required for inclusion in the present study, with 13 years being chosen as the lower limit due to TT-TG measurement stabilization by this age [20]. Two blinded board-certified radiologists then reviewed the MR images of each subject to assess for the presence of meniscal and cruciate tears. Complete meniscal tears (grade 3) were included as were partial or completely torn cruciate ligaments; however, further delineation regarding the specific type of tear (radial, longitudinal, bucket handle, etc.) was not recorded beyond the presence or absence of a tear. The subjects were assigned either to the control group (anatomically intact meniscal and cruciate ligaments) or IKD (meniscal tears, cruciate tears, or a combination of both) based upon MR findings. Subjects were excluded if they had a history of patellar subluxation/dislocation. The presence of severe osteoarthritis (Kellgren and Lawrence, grade 3 or above) or chondromalacia of the patellofemoral joint additionally led to exclusion, as was any unreadable MRI, due to artifact or lack of adequate visualization of the patellar tendon insertion onto the tibial tubercle. Of the 189 MRIs that met the inclusion criteria for age and non-traumatic mechanism of injury, 141 were devoid of the exclusion criteria, thus defining the study population. The control group was comprised of subjects with diagnostically normal MRIs, and the IKD group with those containing meniscal or cruciate tears. Once subdivided into the control and IKD group, the MR images were re-anonymized prior to measurement of the TT-TG and PT-PCL distances by two independent observers, one board-certified radiologist and one fourth-year medical student.

### MR technique and image analysis

MRI images were obtained using a Siemens Avanto 1.5 Tesla scanner. The knees were imaged in an Invivo 8 channel receive only knee coil. The knees were slightly flexed up to 15°. The axial images used to measure the knee anatomy were fat-suppressed proton density fast spin echo sequence with a field of view of 160 mm, matrix 384 × 512, slice thickness of 3 mm, gap of 0.6 mm, bandwidth of 181 Hz/pixel, repetition time (TR) 2600 ms, and echo time (TE) 12 ms. The scans were analyzed on a Centricity PACS workstation (General Electric Healthcare). MRI-based diagnosis of each study subject was determined independently by two board-certified radiologists, and subjects were sorted into control and IKD groups when a diagnostic agreement was reached.

**Fig. 1:**
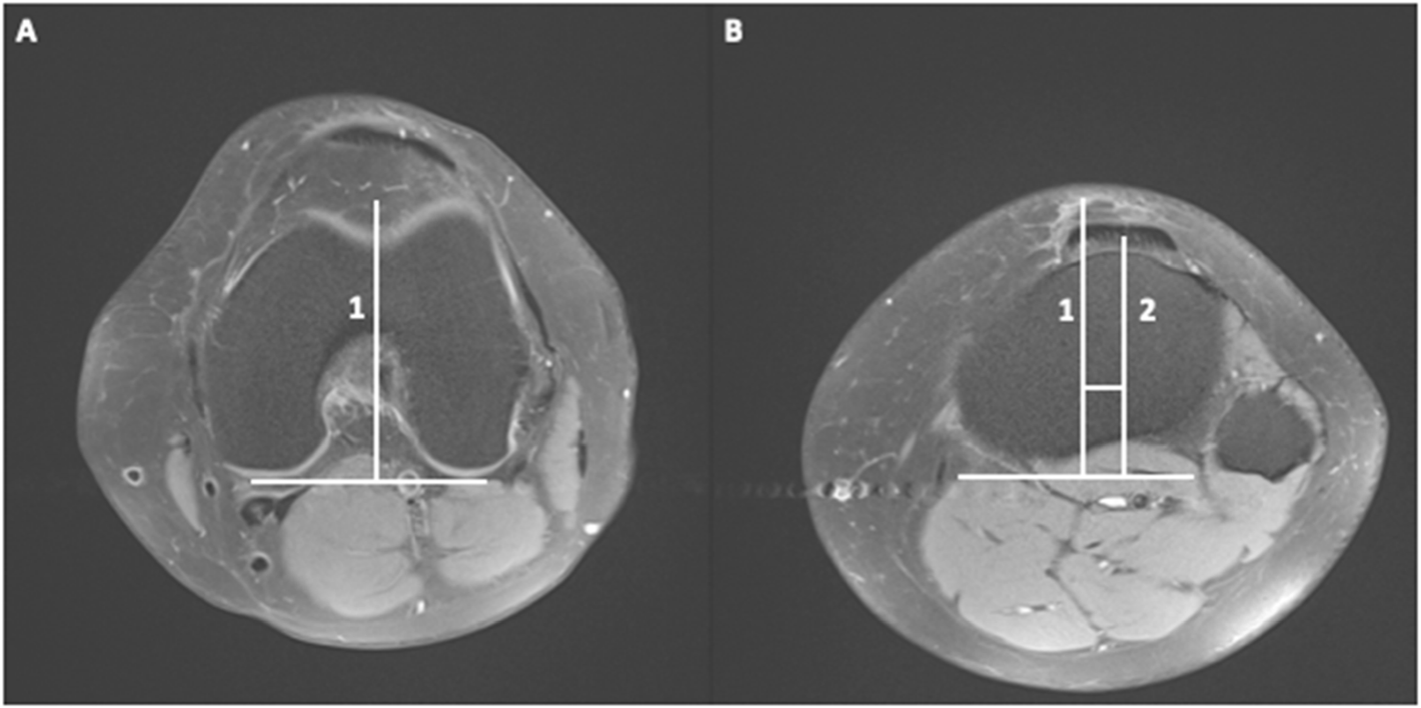
PD MR images of a 34-year-old healthy male subject. **a** Line 1 was drawn through the deepest cartilaginous point of the trochlear groove to form a 90° angle with the posterior condylar line. **b** Line 2 was drawn in parallel to line 1 from the center of the patellar tendon (closest to the tibial tubercle) extending to the original posterior condylar line. The distance between lines 1 and 2 represents the TT-TG

**Fig. 2:**
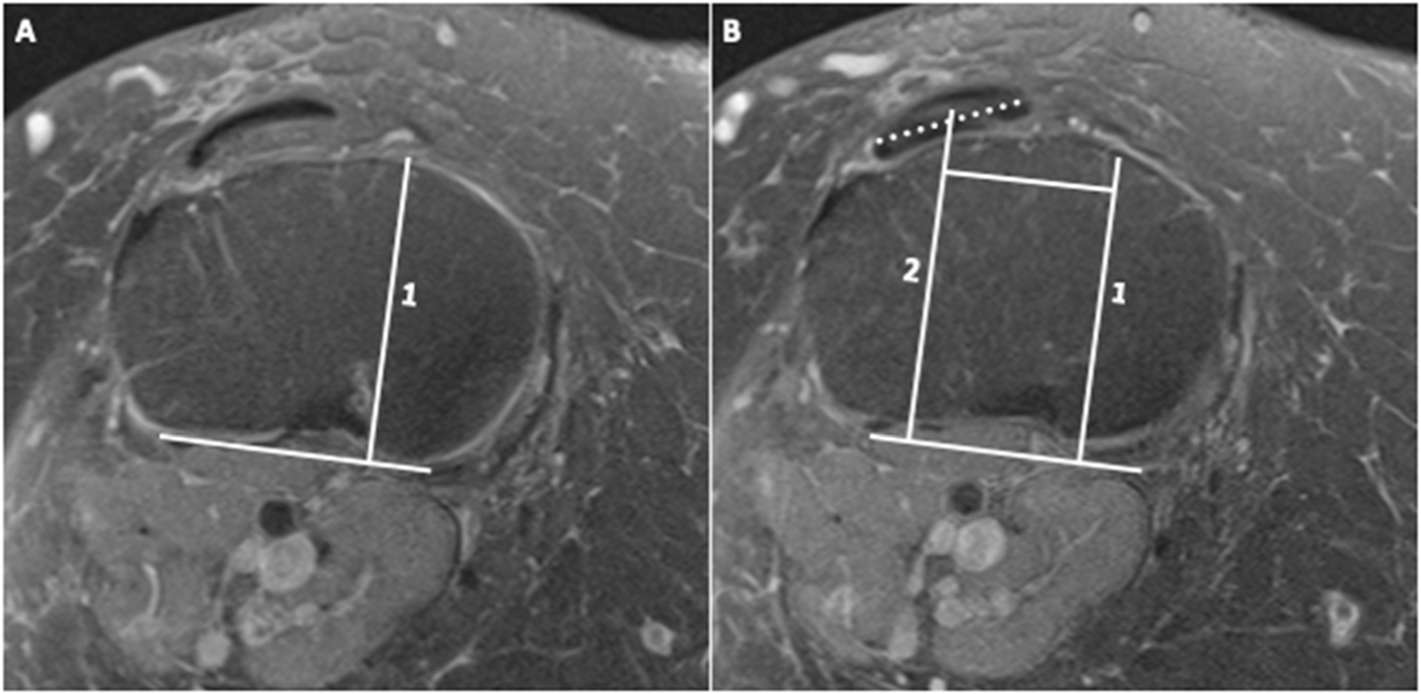
PD MR images of a 32-year-old female subject. **a** Line 1 was drawn through the medial border of the posterior cruciate ligament to form a 90° angle with the posterior tibial condylar line. **b** Line 2 was drawn in parallel to line 1 from the center of the patellar tendon (closest to the tibial tubercle) extending to the original posterior condylar line. The distance between lines 1 and 2 represents the PT-PCL

TT-TG and PT-PCL measurements were performed independently on axial fat-suppressed PD TSE MR images by one board-certified radiologist with over 10 years of experience and one fourth-year medical student, who were both blinded to the MRI diagnostic findings. Measurements were performed following the methods described by Schoettle et al. for the TT-TG (Fig. 1) and Marquez-Lara et al. for the PT-PCL (Fig. 2). The TT-TG measurement was taken by drawing lines from each respective landmark perpendicular to the posterior femoral condylar line with this methodology being repeated for the PT-PCL in relation to the posterior tibial condylar line. Each observer was blinded to the MRI findings as well as to the measurement recorded by their counterpart and both observers measured on the same workstation. Following the completion of the measurements, the study population was re-sorted into control (anatomically normal) and IKD groups. Additionally, the IKD group was further subdivided into those with the presence of isolated meniscal tear, isolated cruciate tear (complete or partial), or a combination of both serving as the experimental group for statistical analysis.

### Statistical analysis

The number of subjects needed in the present study to detect a statistically significant difference in TT-TG distance between groups was performed in a similar fashion to the method described by Saper [13]. Saper et al. found that the mean (SD) difference in TT-TG distance between the ACL deficient and control study groups was 1.63 (3.46) for a Cohen’s d effect size of 0.47. To achieve 80% power assuming an alpha of 5% via two-sided testing and an effect size of *d* = 0.47 would require *n* = 73 per group or *n* = 146 overall subjects. A power of 79% was reached in the present study.

Study data was imported into SPSSv25.0 (IBM Corp.) software. Subjects were classified based on presentation with internal knee derangement (IKD) or not (control). The primary outcome variable, TT to TG distance, was blindly measured by two independent raters and the two-way mixed-effects intraclass correlation model (ICC (2, k)) was used to measure the absolute agreement of the two raters. The ICC (2, k) with a 95% confidence interval fell within 0.75 to 1.00; thus, the two raters were deemed in agreement and their values were averaged to a single value. A similar procedure was performed for the secondary measure, PT-PCL distance, with ICC(2,k) value indicating high reliability hence both rater measurements were also averaged into a single metric value.

TT-TG and PT-PCL distance (mm) were compared between the two study groups, IKD and control, using an independent samples Student’s *t* test since the sample size is large enough to invoke the central limit theorem to justify normality. In the presence of overall significant differences, Tukey post hoc HSD tests were performed to compare IKD subgroups to control. Age was also compared between the two study groups and compared for mean equality. Gender was also compared for distributional equality between the two study groups via Pearson chi-square test. A significant gender imbalance resulted in the use of a multivariate regression model with factors for study group and gender. Thus, the primary analysis for inference purposes is the comparison between study groups after adjustment for gender.

## Results

**Fig. 3:**
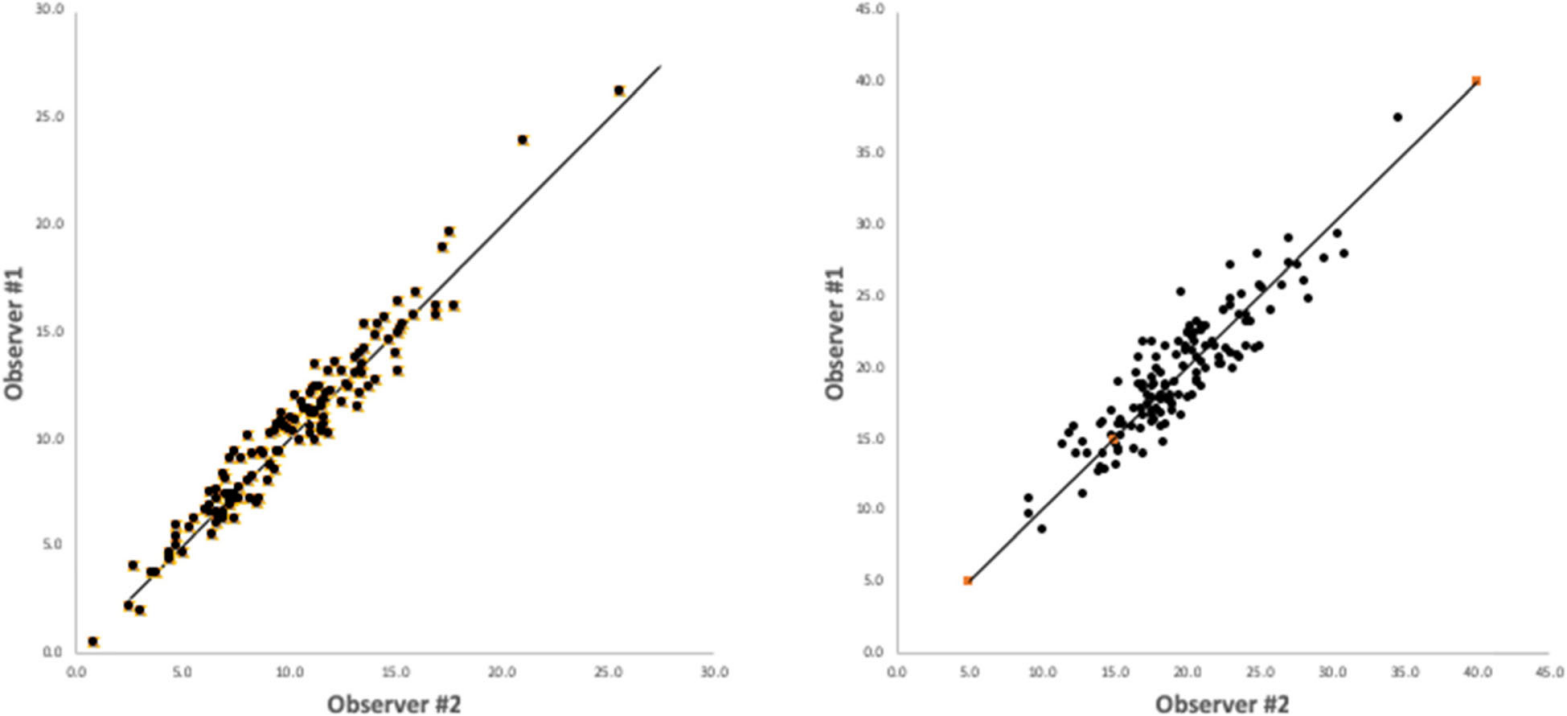
TT-TG (left) and PT-PCL (right) distances were assessed as having excellent reliability via a two-way ICC (2, k) mixed model for absolute mean agreement value of 0.985 (95% CI, 0.979–0.990) and 0.945 (95% CI, 0.923–0.961), respectively

**Fig. 4:**
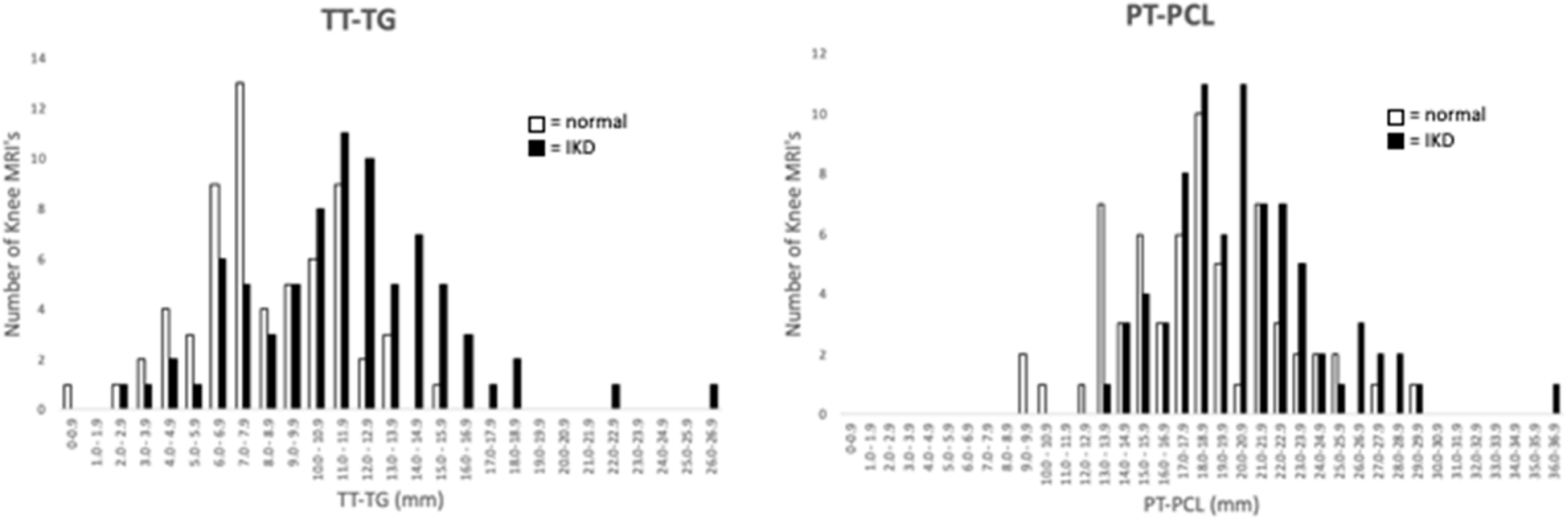
Histogram of TT-TG (left) and PT-PCL (right) measurements

The demographic characteristics for each group are shown in Table 1. Male gender was over-represented in the IKD group (75.6% versus 54.0% control, *p* = 0.007); however, mean age was comparable (33.4 years in IKD vs. 32.9 years in control, *p* = 0.771) as was knee laterality (*p* = 0.182). The two independent, blinded rater measurements of TT-TG and PT-PCL distances were assessed as having excellent reliability via a two-way ICC (2, k) mixed model for absolute mean agreement value of 0.985 (95%CI 0.979–0.990) and 0.945 (95% CI, 0.923–0.961) for the TT-TG and PT-PCL measurements respectively and therefore averaged (Fig. 3). The TT-TG and PT-PCL data are shown in Fig. 4. The mean TT-TG distance averaged across the two raters in the IKD group was 11.5 mm (95% confidence interval [CI], 10.6–12.4), compared to 8.3 mm (95% CI, 7.6–9.0) in the control group (*p* < 0.001). A significant difference was also observed between the mean PT-PCL distance in the IKD group and the control group (20.6 mm versus 18.2 mm, *p* < 0.001).

**Table 1:** Demographic data

	Control (*n* = 63)	IKD (*n* = 78)	*p* value*
Age (years)			0.771
Mean (SD)	32.9 (11.41)	33.4 (10.18)	
Gender—*n* (%)			0.007
Male	34 (54.0)	59 (75.6)	
Female	29 (46.0)	19 (24.4)	
Laterality—*n* (%)			0.182
Left	26 (41.3)	41 (52.6)	
Right	37 (47.4)	37 (47.4)	

*p value for the mean age comparison via independent sample Student’s *t* test. *p* values for categorical comparisons via Pearson chi-square tests

Among the IKD group, there were 51 meniscal tears, 12 cruciate ligament tears, and 15 tears with a combination of meniscal and cruciate findings (Table 2). There was no significant difference in mean TT-TG and PT-PCL distance between IKD subgroups (*p* = 0.165 and *p* = 0.233, respectively); however, each subgroup was significantly greater in TT-TG distance than control after Tukey HSD pairwise post hoc testing (*p* < 0.004 for each). Each IKD subgroup was significantly greater than control (*p* < 0.05 for each) in PT-PCL distance with the exception of the meniscal tear subgroup (*p* = 0.069).

**Table 2:** MRI measurements of the TT-TG and PT-PCL distances

	Control (*n* = 63)	IKD (*n* = 78)	*p* value*
TT-TG distance (mm)			< 0.001
Mean (SD)	8.3 (2.94)	11.5 (4.07)	
Subgroup TT-TG distance (mm)			
Meniscal tears (*n* = 51)		10.9 (3.29)	0.001
Cruciate ligament tear (*n* = 12)		12.2 (4.13)	0.004
Both meniscal and cruciate ligament tears (*n* = 15)		13.0 (5.90)	< 0.001
PT-PCL distance (mm)			< 0.001
Mean (SD)	18.2 (4.22)	20.6 (3.94)	
Subgroup PT-PCL distance (mm)			
Meniscal tear (*n* = 51)		20.0 (3.67)	0.069
Cruciate ligament tear (*n* = 12)		21.5 (3.37)	0.044
Both meniscal and cruciate ligament tears (*n* = 15)		21.7 (5.01)	0.014

*p value for mean TT-TG comparison via independent sample Student’s *t* test. *p* values for individual subgrouped mean comparisons to control via Tukey HSD tests

A multivariate regression model was determined separately for average TT-TG and PT-PCL distance with predictive factors for gender and IKD. IKD was a significant factor in each model even after adjustment for gender with an additional 3.016 mm of TT-TG length (*p* < 0.001) and 2.600 mm of PT-PCL (*p* < 0.003) length associated with IKD. Further, after ROC analysis was applied using the maximum Youden’s J index to determine the optimal cut-off, it was determined that TT-TG distances above 12 mm were strongly associated with the presence of IKD, with only four out of 39 cases (10.3%) having an internally intact knee.

## Discussion

The TT-TG and PT-PCL measurements are clinically useful tools to quantify the degree of patellar lateralization and for use in planning corrective management of severe malalignment. However, there is a lack in consensus regarding which distances constitute the normal range and the reporting of normative data has been highly variable. After controlling for the presence of internal knee derangements, including meniscal and cruciate tears, our study found the anatomically normal range for the TT-TG in symptomatic patients to be 8.3 mm (95% CI, 7.6–9.0). This falls within the lower limit of what has previously been reported [2, 12–15], and it approximates the normal ranges reported by Pandit (9.0–11.0 mm) [14] and Wittstein (8.8–10.0 mm) [15] which represent this lower limit. Variation in knee position may further account for discrepancies between these ranges and the normal range of the present study [17]. Given that Pandit and Wittstein performed MRI acquisition with knees in full extension (which leads to an overestimation of the TT-TG) and that the present study protocol was performed with knees in 15° of flexion, it is likely that the mean values in the aforementioned studies would align even closer with the normal range in this study if identical knee protocols were followed.

Further, in comparison to the normal range of 10.4 mm (95% CI, 9.64–11.24) reported by Saper, [16] where a 25° of knee flexion protocol was used, the control group in the present study still underestimated this mean by almost 2 mm. Yet when comparing the IKD cruciate injury subgroup (12.2 mm, *p* < 0.004) in the present study to the ACL deficient group (12.95 mm, *p* < 0.005) in the Saper study (the primary pathology accounted for in the study), the results closely approximate each other. These findings could be explained by the confounding presence of meniscal tears within the Saper study, leading to a type II error in the control group. With meniscal tears in their control group, the normal range was overestimated. By accounting for meniscal tears in the present study, the data reveal a greater difference (3.27 mm) between the internally deranged and anatomically intact knees than what was previously reported by Saper et al. (1.63 mm) [16]. This difference is both greater and more significant than what was previously reported, therefore supporting the association of increased TT-TG distances with both meniscal and cruciate internal knee derangements.

However, within the context of the PT-PCL measurement in the present study, statistical significance was lost (*p* < 0.069) in this meniscal tear subgroup. Changes in knee positioning or a relative decrease in interrater reliability due to the increased challenge of picking the same slice across observers while measuring the PT-PCL measurement (as compared to the TT-TG measurement) are both plausible reasons for this occurrence. Despite these results, the combination derangement subgroup (meniscus tears and ACL tears) had the highest mean across the TT-TG (13.0 mm) and PT-PCL (21.7 mm) measurements. Across the TT-TG and PT-PCL measurement, the meniscal tear subgroup had the lowest mean, followed by the cruciate subgroup, and finally by the combination derangement group which had the highest TT-TG and PT-PCL overall. Collectively and individually, these averages were well above the normal ranges for the TT-TG and PT-PCL measurements and well below the patellar dislocation rage of 15–20 mm for the TT-TG measurement, supportive of the study hypothesis

Although further mechanistic knee studies may be helpful, we believe this new association between increased TT-TG distances and internal knee derangements may be explained by the presence of an increased lateral stress applied across the knee joint during dynamic movement. With the increasing lateralized pull of the quadriceps across the knee (denoted by a widened TT-TG), an imbalance may arise between the stabilization of rotational vectors at work within the joint causing increased imbalance during static and dynamic rotation. It is hypothesized that over time, this imbalance is what may predispose subjects with increased TT-TG and PT-PCL distances to tears both within the menisci and cruciate ligaments. Once the lateralization reaches a critical threshold value of 15–20 mm; however, the risk of patellar dislocation exponentially increases and thus becomes the overarching pathology observed. While further studies are needed to explore this hypothesized difference between patients with normal and wide TT-TG and PT-PCL distances, the present study provides critical findings from which to investigate these relationships.

There are several limitations to our study. The retrospective nature of this study limited our ability to explore the number of control patients that would eventually convert to the meniscal or cruciate injury groups as well as those who might eventually convert to a patellar dislocation. The post hoc power of 79% for our primary comparison was close to our minimal goal of 80%, and it was decided rather than introduce problems with multiplicity and potential inclusion biases to not expand enrollment. In the present study, it is also worth noting that while all subjects in the control group had anatomically normal MRIs, many received scanning due to complaints of knee pain or knee discomfort, for which no anatomical explanation was ultimately found. Although a future study with asymptomatic and anatomically normal patients would bolster our current findings, the cost-prohibitive and prospective nature of the study was beyond our scope, as was delineating which specific types of meniscal tears occurred more commonly, due to our relatively small study population.

Another challenge of this study was found in choosing which MR slice best depicted the patellar tendon/tibial tubercle junction and the medial border of the PCL [21], with the latter landmark being by far the most challenging part of the study. While the interrater reliability was excellent between the two blinded rater measurements (0.985 and 0.945 for TT-TG and PT-PCL, respectively), lateralized or medialized insertion of the patellar tendon on the tibial tubercle, variable tibial tubercle morphology, inadequate distal imaging acquisition, intrasubstance signal within the PCL, and variability in observer choice of each landmark were all confounding factors in the reproducibility of the measurement.

Gender imbalance within the IKD group (59 males vs 19 females) may also have affected our results. Given that females have an increased relative risk of ACL rupture in non-contact injuries, [22] likely due to an increased TT-TG distance as compared to their male counterparts, it is possible that our data underestimates the true IKD range. In the control group, this was not an issue as gender was equally balanced (34 males and 29 females). Finally, neither group was matched for BMI, tibial plateau width, or patient height. This data was not readily available, and thus, it was omitted. However, it would be important in future prospective studies to record this data to eliminate confounding factors.

## Conclusion

In conclusion, this study adds to the literature regarding normative TT-TG values and additionally draws attention to a previously unreported association between increasing TT-TG and PT-PCL distances and internal knee derangements. While the TT-TG measurement has been scrutinized due to a dependence on knee positioning, the present study utilizes the PT-PCL measurement as a secondary measurement to the TT-TG, given its consistency irrespective of knee positioning. In the present study, the IKD group falls in a previously unidentified region between the anatomically normal control group cases and the previously reported range for patellar dislocation cases. Even after adjustment for gender, an additional 3.016 mm of TT-TG length and 2.600 mm of PT-PCL length was associated with IKD. Further, it was determined that TT-TG distances above 12 mm were strongly associated with the presence of IKD, with only four out of 39 cases (10.3%) having an internally intact knee. While the TT-TG is only one contributing factor, a distance greater than the 12 mm threshold appears to be statistically associated with internal knee injuries in this study group.

## Data Availability

The datasets used and/or analyzed during the current study are available from the corresponding author on reasonable request.

## Abbreviations

IKD: Internal knee derangements
PT-PCL: Patellar tendon-posterior cruciate ligament
TT-TG: Tibial tubercle-trochlear groove
